# Psychometric Properties of the Spanish Version of the Patient Health Questionnaire-9 (PHQ-9) in Mexican Adolescents

**DOI:** 10.1192/j.eurpsy.2026.11161

**Published:** 2026-07-31

**Authors:** L. Díaz-Castro, C. Díaz de León-Castañeda

**Affiliations:** 1 Department of Epidemiological and Psychosocial Research, National Institute of Psychiatry Ramón de la Fuente Muñiz; 2 Researchers for Mexico, Secretariat of Science, Humanities, Technology and Innovation (SECIHTI), Mexico City, Mexico

## Abstract

**Introduction:**

The Patient Health Questionnaire-9 (PHQ-9), in particular, is widely recommended due to its brevity, ease of use, and strong psychometric validity for screening, diagnosing, and monitoring the presence and severity of depressive symptoms, including its use in adolescents.

**Objectives:**

This study aimed to evaluate the psychometric properties of the Spanish version of the PHQ-9 in a representative sample of adolescents from San Luis Potosí, Mexico, to support its use as a reliable screening tool for depressive symptoms in this population

**Methods:**

A total of 768 adolescents participated in the study. Confirmatory Factor Analyses (CFA) were performed to assess both one-factor and two-factor models, using estimation methods appropriate for ordinal data. Internal consistency was evaluated using both Coefficient Alpha (α) and Omega (ω). Measurement invariance across sex was tested, and concurrent validity was examined through correlations with the Generalized Anxiety Disorder-7 (GAD-7) scale.

**Results:**

CFA results demonstrated that both the one-factor and two-factor models provided a very good fit to the data. Internal consistency was acceptable to excellent across models (α = 0.779 to 0.896). Strong measurement invariance by sex was confirmed for both models. Furthermore, a strong positive correlation was found between PHQ-9 and GAD-7 total scores (r = 0.812), supporting the scale’s convergent validity.

**Conclusions:**

The Spanish version of the PHQ-9 exhibits robust psychometric properties in Mexican adolescents, including solid factorial validity, reliability, measurement invariance, and convergent validity. These findings support its use as an effective tool for the assessment of depressive symptoms in this population.

**Disclosure of Interest:**

None Declared

